# New *Brucella* variant isolated from Croatian cattle

**DOI:** 10.1186/s12917-021-02833-w

**Published:** 2021-03-20

**Authors:** Silvio Spicic, Maja Zdelar-Tuk, Claire Ponsart, Rene S. Hendriksen, Irena Reil, Guillaume Girault, Pimlapas Leekitcharoenphon, Vesna Rukavina, Martina Rubin, Luca Freddi, Sanja Duvnjak

**Affiliations:** 1 Department of Bacteriology and Parasitology, Laboratory for Bacterial Zoonosis and Molecular Diagnostics of Bacterial Diseases, Croatian Veterinary Institute, Savska street 143, Zagreb, Croatia; 2 French Agency for Food, Environmental & Occupational Health Safety (ANSES), Bacterial Zoonoses Unit - Animal Health Laboratory, National & OIE/FAO Animal Brucellosis Reference Laboratory, EU Reference Laboratory for Brucellosis, 14 rue Pierre et Marie Curie, Maisons-Alfort, Cedex France; 3 Technical University of Denmark, National Food Institute, Research Group for Genomic Epidemiology, Kemitorvet, Building 204, 2800 Kgs. Lyngby, Denmark; 4 Ministry of Agriculture, Veterinary and Food Safety Directorate, Veterinary Office Sisak, Branch Office Glina, Trg bana Josipa Jelačića 2, 44 400 Glina, Croatia; 5 Ministry of Agriculture, Veterinary and Food Safety Directorate, Department for Veterinary Epidemiology, Planinska 2a, 10000 Zagreb, Croatia

**Keywords:** *Brucella melitensis*, Variant, Cattle, Eradication, Croatia

## Abstract

**Background:**

A novel *Brucella* strain closely related to *Brucella* (*B*.) *melitensis* biovar (bv) 3 was found in Croatian cattle during testing within a brucellosis eradication programme.

**Case presentation:**

Standardised serological, brucellin skin test, bacteriological and molecular diagnostic screening for *Brucella* infection led to positive detection in one dairy cattle herd. Three isolates from that herd were identified to species level using the Bruce ladder method. Initially, two strains were typed as *B. melitensis* and one as *B. abortus*, but multiplex PCR based on IS*711* and the Suis ladder showed that all of them to belong to *B. melitensis,* and the combination of whole-genome and multi-locus sequencing as well as Multi-Locus Variable numbers of tandem repeats Analysis (MLVA) highlighted a strong proximity within the phylogenetic branch of *B. melitensis* strains previously isolated from Croatia, Albania, Kosovo and Bosnia and Herzegovina. Two isolates were determined to be *B. melitensis* bv. 3, while the third showed a unique phylogenetic profile, growth profile on dyes and bacteriophage typing results. This isolate contained the 609-bp *omp31* sequence, but not the 723-bp *omp31* sequence present in the two isolates of *B. melitensis* bv. 3.

**Conclusions:**

Identification of a novel *Brucella* variant in this geographic region is predictable given the historic endemicity of brucellosis. The emergence of a new variant may reflect a combination of high prevalence among domestic ruminants and humans as well as weak eradication strategies. The zoonotic potential, reservoirs and transmission pathways of this and other *Brucella* variants should be explored.

**Supplementary Information:**

The online version contains supplementary material available at 10.1186/s12917-021-02833-w.

## Background

Brucellosis in cattle, which can be caused by *B.abortus*, *B. melitensis* and *B. suis* [1], can significantly impact productivity on beef and dairy farms, and it poses zoonotic risk to humans, in whom infection can cause severe illness. The last reported *B. abortus* infections in cattle in Croatia occurred in 1964, while *B. melitensis* infections in cattle were reported in 2008 in herds kept with infected sheep [2] and in 2019 in herds kept with infected goats [3]. Since 2011, Croatia has conducted a brucellosis eradication programme in cattle according to European Directive 64/432/EC. All animals older than 12 months are tested annually using the Rose Bengal test (RBT), and positive animals are further tested using complement fixation (CFT), as well as indirect and competitive ELISAs. Depending on the epidemiological situation, seropositive animals are tested using a brucellin skin test, tissues and organs (head, mammary and genital lymph nodes, uterus, spleen, udder, fetal membranes) and stomach contents, spleen and lung from foetuses are collected at slaughterhouse for bacteriological testing.

Within this eradication programme, potential *Brucella* isolates are identified to genus and species levels using a combination of classical biotyping, multiplex PCR and molecular genotyping methods, which can include Multi-Locus Variable number of tandem repeats Analysis (MLVA), Multi-Locus Sequence Typing (MLST) [1, 4–7] and Whole-Genome Sequencing (WGS).

Here we describe the identification of a dairy cow herd with brucellosis within the framework of the Croatian eradication programme. The disease was attributed to infection with *B. melitensis* bv. 3, except in one cow infected with a *B. melitensis* variant difficult to identify using standard classical and molecular methods. The emergence of this novel strain points to ongoing *Brucella* evolution in the western Balkans area, which may be due to the appearance of new reservoirs or vectors forced strain mutation and poor efficacy of eradication measures. Future studies should explore new reservoirs and zoonotic significance for this and other potential new *Brucella* variants in the region.

## Case presentation

During routine annual testing within the national eradication programme, a dairy cow herd (12 cows, 7 heifers and 4 calves) with brucellosis was identified in October 2018 on a farm in the Croatian village of Katinovac (45°14′30.6″N 15°55′31.8″E), close to the northern border with Bosnia and Herzegovina. At the time of testing, animals were healthy and showed no clinical signs of brucellosis. Other animal species were not present on farm. A total of 19 animals aged 1 year and older were tested using the RBT. A total of 10 animals were identified positive by RBT and indirect ELISA, and 7 of these animals were also positive by the CFT and competitive ELISA. In November 2018, 19 animals older than 1 year were tested on the brucellin skin test. Positive reaction on brucellin was found in 5 previously seropositive cows, which were sent to the slaughterhouse, where samples were collected and analysed bacteriologically. During the post mortem inspection visible lesions were not recorded.

Epidemiological investigation showed that the Croatian herd had been brucellosis-free since 2013, and no new animals had been introduced since 2008. Since 2016, the farm practised artificial insemination with no recent history of abortions. The farm owner denied contact with other sheep or cattle herds and indicated that the herd was kept on pastures bordering Bosnia and Herzegovina. Their water source was the river Glina, a natural borderline in this area.

*Brucella* strains were isolated from milk and various tissues from three cows that were found without any clinical symptoms. The strains were classically biotyped as described [4] based on CO_2_ requirement, H_2_S production, oxidase and urease activity, growth on dyes, lysis by phages and agglutination with monospecific sera (Tables 1 and 2). This biotyping was performed at the Croatian National Reference Laboratory (Zagreb) and European Reference Laboratory (Maisons-Alfort, France). All three isolates could grow without CO_2_, they produced H_2_S and expressed oxidase, and they hydrolysed urea. They did not grow on basic fuchsin medium, and they triggered agglutination of anti-A and anti-M sera. These findings are consistent with *B. melitensis* bv.3. However, isolate 7 was lysed by Tbilisi phages at 10^4^ routine test dilution (RTD).

**Table 1:** Results of classical biotyping tests of *Brucella* isolates

Strain	CO_2_	H_2_S	Growth on		Lysis with			Agglutination
			thionin	fuschin	Tbilisi phages RTD/10^4^RTD	Weybridge phages	Izatnagar1 phages	
B.melitensis 16M	–	–	+	+	−/−	–	–	M+
B.abortus 544	+	+	–	+	+/+	+	+	A+
Isolate 6	–	–	+	–	−/−	+	+	M+, A+
Isolate 7	–	–	+	–	−/+	+	+	M+, A+
Isolate 11	–	–	+	–	−/−	+	+	M+, A+

*A* Antiserum A, *M* Antiserum M, *RTD* Routine test dilution

**Table 2:** Molecular identification and genotyping of *Brucella* isolates

Method(s)	Reference	Result
PCR AMOS	Bricker &Halling, 1993	Isolates 6, 7, 11: *Brucella melitensis*
Bruce ladder	Lopez-Goni et al. 2008	Isolate 6: *Brucella melitensis* Isolate 7: *Brucella abortus* Isolate 11:*Brucella melitensis*
PCR omp31	Vizcaino et al., 1997	Isolate 6:positive Isolate 7: negative Isolate 11: positive
Suis ladder	Lopez-Goni et al., 2011	Unique pattern for all *B. melitensis* strains
MLVA 16 (Bruce06–08 - 11 - 12 - 42 - 43- 45 - 55, 18–19 - 21, 04–07 - 09 - 16 - 30)	La Fleche et al., 2006; Al Dahouk et al., 2007	Isolate 6: 1–5–3-13-3-2-3-2-4-41-8-5-4-3-7-6 Isolate 7: genotype (1–5–3-13-2-2-3-2-4-41-8-5-4-3-7-6), consistent with *B. melitensis*
MLST 9 (gap - aroA - glk - dnaK - gyrB - trpE - cobQ - int-hyp (orf1)-omp25)	Whatmore et al., 2007	Isolates 6 and 7: sequence type 8, 3–2–3-2-1-5-3-8-2; consistent with *B. melitensis*
WGS/wgSNP	Illumina NexteraXT guide (no. 150319425031942), following protocol revision C	DDBJ/ENA/GenBank under accession numbers CVI_6 ChI CP058599, CVI_6 ChII CP058600, CVI_7 ChI CP058597, and CVi_7 ChII CP058598

Species was determined using multiplex PCR based on the Bruce ladder [8] and the “AMOS” method [9], followed by the Suis ladder [10] and another PCR based on detection of the *omp*31 gene [11]. Isolates 6 and 11 gave results consistent with *B.melitensis,* but isolate 7 lacked *omp*31 gene sequences tested in the Bruce ladder, suggesting that it was *B.abortus*.

To confirm the identification of isolates 6 and 11 as well as to complete the identification of isolate 7, we performed MLVA based on 16 loci [5, 6] in the following order: *panel 1, Bruce*06 - *Bruce*08 - *Bruce*11 - *Bruce*12 - *Bruce*42 - *Bruce*43- *Bruce*45 - *Bruce*55; *panel 2a, Bruce*18 - *Bruce*19 - *Bruce*21; and *panel 2b, Bruce*04 - *Bruce*07 - *Bruce*09 - *Bruce*16 - *Bruce*30. *B. melitensis* 16M was used as the reference strain for comparison and verification of test quality. In addition, MLST was performed based on 9 loci [7]: *gap - aroA - glk - dnaK - gyrB - trpE - cobQ - int-hyp* (*orf1*)-*omp25*.

Moreover, isolates 6 and 7 were subjected to whole-genome shotgun sequencing using the Illumina NexteraXT system (protocol 150,319,425,031,942, revision C), which has been deposited in DDBJ/ENA/GenBank under accession numbers CVI_6 ChI CP058599/CVI_6 ChII CP058600 and CVI_7 ChI CP058597/CVI_7 ChII CP058598. A phylogenetic tree was generated using Bioumerics 7.6.3 (Applied Maths, BioMérieux). A set of *B. melitensis* genomes was retrieved from public databases (NCBI and PATRIC) and numbered in table (see Additional file 1). Sequencing reads were simulated for each genome using ART and all reads were mapped against a chimeric genome of *B. melitensis* 16M genome. Eight *B. abortus* genomes were used as outgroup. The SNPs obtained were then filtered (20X of absolute coverage, 10 bp inter-SNP distance, ambiguous and unreliable bases were removed, repeated elements removed) and a maximum parsimony tree was generated from these SNPs. The tree is represented with a logarithmic scale (see Additional files 2 and 3). This sequencing also revealed that isolate 7 contained a 609-bp *omp*31 sequence also present in isolate 6, but not the 723-bp *omp*31 sequence present in isolate 6. Based on classical and molecular methods, we assigned *Brucella* isolates 6 and 11 as *B. melitensis* bv. 3, while isolate 7 appeared to be a novel *B. melitensis* variant.

## Discussion and conclusions

Human brucellosis, considered one of the most dangerous zoonoses, is most often caused by *B. melitensis* and less often by *B. abortus* or *B. suis*. The disease is endemic to the Mediterranean in general and the Balkan peninsula in particular [12–16]. Nevertheless, bovine brucellosis cases are sporadic and infrequent in Croatia, with the only recent reports limited to instances of transmission from sheep and goats on the same farms [2, 3]. The present case is the first recent report of brucellosis in Croatia that cannot definitively be attributed to contacts with other infected animal species.

This work highlights the need for continuing vigilance and research into potential *Brucella* reservoirs and spreading pathways. The disease in the present report may easily have come from Bosnia and Herzegovina, because herds on both sides of the border often share pastures, and illegal migrations are common which is documented throughout complete border line between countries [2, 3, 17]. Bosnia and Herzegovina has conducted a vaccination programme to control brucellosis in small ruminants since 2009, yet incidence of the disease remains high in animals and humans [18], and has even been increasing since 2012 [3]. This lack of efficacy is likely due largely to non-compliance with vaccination programmes [18], which can also foster the emergence of new *Brucella* strains [19].

We were unable to identify isolate 7 using classical microbiological methods [4] which are based on phenotype. This suggests that classical methods may not be well suited for characterising new *B. melitensis* strains in brucellosis-endemic regions. In fact, we were able to unambiguously identify the three isolates only by combining MLVA, MLST and whole-genome sequencing. These techniques showed our strains to be phylogenetically related to strains circulating in Croatia as well as Bosnia and Herzegovina [15, 17]. In particular, MLVA typing allowed us to assign a unique 16-digit code to the novel isolate 7, based on differences from the *Bruce*42 locus. Isolates 6 and 7 were assigned to the previously reported sequence type 8, related to *B. melitensis* strains circulating in Turkey, Kosovo and Macedonia. The two strains CVI_6 and CVI_7 clustered together in a subclade comprising 4 others strains (F9/05 from Turkey, BwIM_XXX_12 from unknown origin, F8/01–155 from Kosovo and BwIM_ALB_46 from Albania). Interestingly, this subclade contains two strains from the Balkan and one from Turkey. Moreover, in a recent paper [20], a strain from Serbia is clustered with the strain of Albania. The 2 strains isolated in this study seem to belong to a clade composed by strains that circulate in the Balkan area (see Additional files 2 and 3). Cross-contamination at the borders with animals can be a reason.

Our findings highlight the need for continuing, even enhanced, efforts to surveillance brucellosis in domestic animals and to research potential *Brucella* reservoirs and transmission pathways to ensure timely detection of zoonotic threats.

## Supplementary Information

**Additional file 1.** Table with strains metadata.

**Additional file 2.** Textual description of phylogenetic tree.

**Additional file 3.** Figure Phylogenetic Tree.

## Data Availability

All data generated during this study are included in this published article. The datasets of WGS genomes generated during the current study are available in the DDBJ/ENA/GenBank repository under accession numbers CP058599-CP058600 and CP058597-CP058598.

## Abbreviations

NCBI: The National Center for Biotechnology Information advances science and health by providing access to biomedical and genomic information (https://www.ncbi.nlm.nih.gov/)
PATRIC: The Pathosystems Resource Integration Center is the all-bacterial Bioinformatics Resource Center (BRC) (http://www.patricbrc.org).
