# Supplementary material for: New Brucella variant isolated from Croatian cattle
Source: BMC Vet Res. 2021 Mar 20;17:126. doi: 10.1186/s12917-021-02833-w (PMC7981855; doi:10.1186/s12917-021-02833-w)
Supplement: Supplementary file 2 — Additional file 2. Textual description of phylogenetic tree. [file 12917_2021_2833_MOESM2_ESM.docx]

Phylogenetic tree legend

The phylogenetic tree represents the phylogeny of a set of *B. melitensis* genomes, including the two Croatian strains, and has been rooted with eight *B. abortus* reference genomes. The two isolates of this study are highlighted with a black arrow (in Additional file Figure Phylogenetic Tree). Each circle represents a *Brucella* genome, and the size of the circles is proportional to the number of isolates. The color code is related to the geographic origin of the strains, i.e. Africa, Asia, Eurasia, Europe, North or South America or Not Reported.
