# Supplementary figures and images for: New Brucella variant isolated from Croatian cattle
Source: BMC Vet Res. 2021 Mar 20;17:126. doi: 10.1186/s12917-021-02833-w (PMC7981855; doi:10.1186/s12917-021-02833-w)

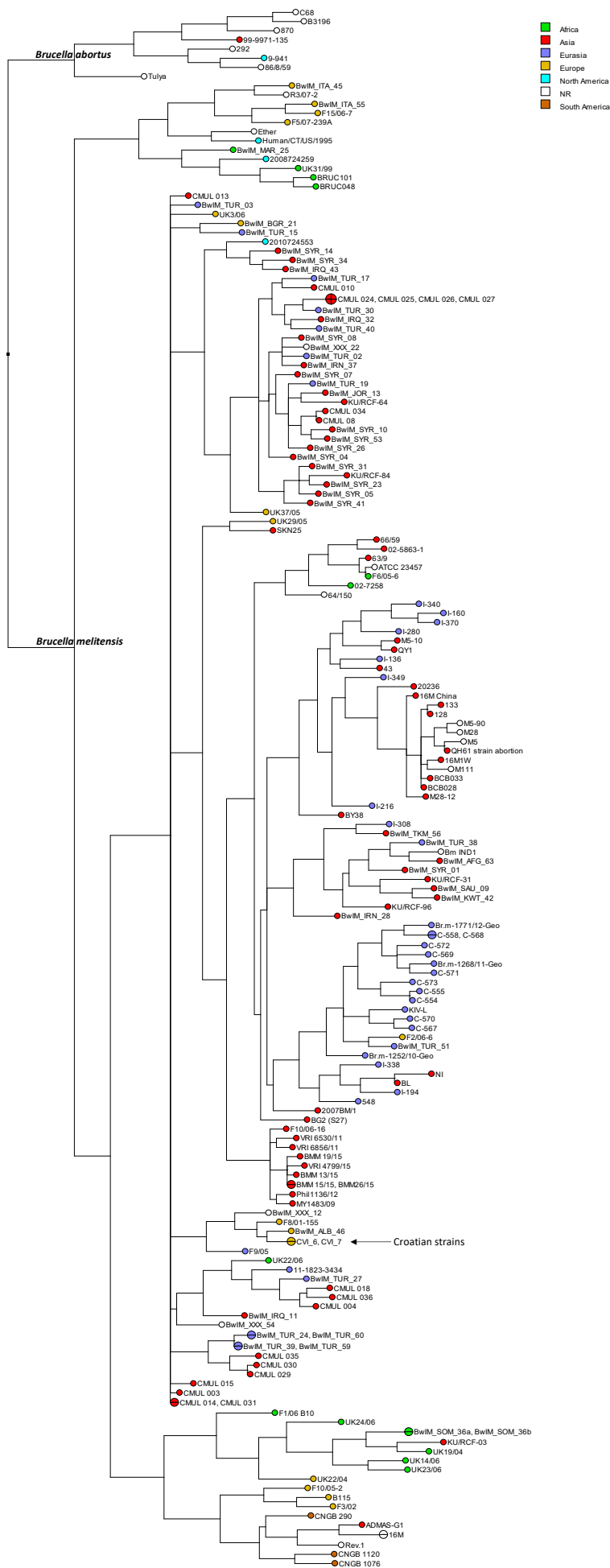

Supplement: Supplementary file 3 — Additional file 3. Figure Phylogenetic Tree. [file 12917_2021_2833_MOESM3_ESM.pdf]
